# Quality of life and mental health outcomes: the role of sociodemographic factors in the Palestinian context

**DOI:** 10.1038/s41598-023-43293-6

**Published:** 2023-09-29

**Authors:** Dana Bdier, Guido Veronese, Fayez Mahamid

**Affiliations:** 1 Psychology and Counseling Department, An-Najah National University, Nablus, Palestine; 2 University of Milano-Bicocca, Milan, Italy

**Keywords:** Psychology, Public health

## Abstract

The Mental health of Palestinians has been described as among the lowest in the world, with over half of Palestinian adults meeting the diagnostic threshold for depressive symptoms and a significant portion of Palestinians experiencing mental distress and anxiety. The aim of the current study was to test the correlation between quality of life (QoL) and mental health outcomes, and the role of sociodemographic variables in predicting mental health outcomes (depression, anxiety, and stress) among Palestinian adults during a challenging period of political conflict. The sample of our study consisted of 957 participants, 283 males and 674 females they were recruited using online methods; online advertisements, e-mail campaigns and social media. Our findings showed that QoL negatively correlated with stress (r = − 0.43, *p* < 0.01), anxiety (r = − 0.46, *p* < 0.01), and depression (r = − 0.47, *p* < 0.05). Moreover, stress positively correlated with anxiety (r = 0.81, *p* < 0.01), and depression (r = 0.89, *p* < 0.01). Finally, anxiety positively correlated with depression (r = 0.82, *p* < 0.01). Results of hierarchical regression analysis to predict stress, anxiety and depression, indicated that QoL, educational level, gender, region of residence, and age explained in a significant way variance in depression, anxiety and stress. Our findings are promising to conduct other studies in order to understand better how current study variables correlate to each other, so appropriate clinical interventions to mitigate the negative effects of depression, anxiety, and stress through enhancing quality of life and positive coping strategies can be developed and implemented by mental health providers.

## Introduction

Quality of life (QoL) can be defined as how individuals evaluate the goodness of different aspects of their lives, including satisfaction with their relationships and work, satisfaction with their life, emotional reaction to life occurrences, and disposition^1^. According to the World Health Organization (WHO), QoL is considered “the individuals’ perception of their position in life in the context of the culture and value systems in which they live and in relation to their goals, expectations, standards, and concerns” (Group^2^, p.28). Based on this definition, QoL can be assessed by exploring individuals’ subjective perceptions about their lives concerning their physical, psychological, social, and economic status^3^.

From an individualistic perspective, quality of life is assessed according to the individuals' social, physical, material, and emotional well-being, development and activity (e.g., choice and control), and their subjective feelings regarding objective life conditions, personal values, and aspirations^4,5^.

In war-affected areas, quality of life is negatively affected due to the long-lasting consequences of conflict, such as changes in social conditions through increased poverty, lack of employment, community violence, inadequate living circumstances, and changes in social networks^6^.

Regarding our study, Palestinians’ quality of life and mental health is expected to be negatively affected due to more than 74 years of military violence. The overall population are still experiencing several traumatic in a war-torn environment characterized by military invasions, detentions, land confiscations, house evictions, and demolitions, being physically injured, loss of loved ones, exposure to an immediate risk of life, and injury of a family member or friend^7–10^.

Furthermore, due to the ongoing military occupation, Palestinians face different challenging life situations, including poverty, lack of employment opportunities, and economic dependence^11^. Violation of justice and equality, territorial fragmentation, building restrictions, cultural pressures, and future insecurity compromise the Palestinians' QoL^12^. Destruction of social networks, fewer positive social outlets due to the restrictions on movement between communities, and lack of recreational facilities are additional burdens for the civil population^13,14^.

According to previous literature, these challenging life conditions and living in the war-affected area were found to undermine the quality of Palestinians’ life, in which one-third of adult Palestinians reported low levels of well-being^15,16^. Low health-related quality of life (physical, psychological, and environmental) was significantly associated with war-related factors, especially mental distress, insecurity and psychological suffering among Palestinian adults^17^. Furthermore, in a study that explored QoL among injured people in the Gaza war, the results showed that their QoL were very low in all domains of QoL^18^.

Human insecurity due to the political conflict, chronic economic constraints, and prolonged exposure to violence were positively associated with depressive symptoms, high depression, and suicidal ideation among Palestinian adults^19–21^. In addition, low levels of quality of life were found to be associated with feelings of hopelessness and depressive symptoms among Palestinians in the West Bank^22^.

Hence, due to the challenging living situations in the Palestinian context, several demographic variables were found to be negatively correlated with quality of life and mental health status, including poverty, being married, and working 15 h or more per day. Studies found that a higher standard of living was associated with high levels of well-being, while individuals living in Palestinian refugee camps reported low levels of well-being^15^. Also, men, older persons, and those less educated were found to have lower levels of QoL among Palestinian adults^16^. Moreover, in a study that examined psychiatric symptomatology characterized by general distress, depression, and anxiety associated with direct and indirect forms of violence exposure among Palestinian young adults, the findings revealed that women reported higher levels of global distress, depression, and anxiety than men^21^. In a literature review that examined the relationship between home demolition policy toward Palestinians and mental health consequences, the results showed that many life stressors such as lack of educational opportunities, low incomes, and a tendency to live in poor housing conditions after and before demolition also played a role in developing severe mental disorders such as depression, stress, and anxiety^23^. Our study drives its importance as it’s the first study which was designed to test the association between current study variables in the Palestinian context, which characterized by a high level of political conflict and mental distress.

Moving from the above-described background, our study sought to test the association between QoL and mental health outcomes, and the role of specific demographic variables (educational level, gender, region of residency, and age) in predicting mental health outcomes in terms of stress, anxiety and depression among Palestinians. Based on prior research^15–23^, study hypotheses were defined: First, QoL would be negatively associated with mental health outcomes operationalized by (depression, anxiety, and stress) among Palestinian adults (H1); Second, educational level, gender, region of residency, and age would predict mental health outcomes(depression, anxiety, and stress) among Palestinian adults (H2); Third, QoL and demographic variables (educational level, gender, region of residency, and age would predict together mental health outcomes (depression, anxiety, and stress) among Palestinian adults (H3).

## Methodology

### Participants

We conducted our study in the West Bank, East Jerusalem and Gaza Strip during a challenging period of political conflict between Israelis and Palestinians. This period witnessed many violent incursions and confrontations between the Israeli soldiers and the Palestinians. Participants of our study were 957, including 283 males and 674 females. The majority, 68.9%, of participants was from urban regions, 19.3 percent were from rural regions, and 11.8 per cent were from Palestinian internally displaced camps. 32.1 held a master’s degree, 54.6 with a Bachelor's degree, and 13.3 with a high school degree. Regarding geographical region, 41.5 participants were from West Bank and 48.5 from the Gaza strip. 1.8 participants aged 18–20, 29.4 aged 26–33, 30.6 aged 34–43, 23.7 aged 41–55, and 14.5 aged more than 51 years.

Participants were recruited using online methods; online advertisements, e-mail campaigns and social media. The sample size for this study was calculated based on 95% CI and 5% margin of error by using the Raosoft software sample size calculator. Based on that, the recommended sample was 957 participants. Inclusion in the study required participants to be (1) Palestinian, (2) living in the West Bank of Palestine, Gaza strip and East Jerusalem, (3) native Arabic speakers, and (4) free from having any neurodevelopmental and mental disorders. We excluded from our study all participants who do not live in the West Bank of Palatine, Gaza strip and East Jerusalem.

### Measures

Following standard methodological recommendations for developing our questionnaires^24^, all items were translated and back-translated from the original English version to Arabic and pilot-tested by a panel of ten Arab professionals recognized as experts in psychology, counselling, and social work. These professionals evaluated the clarity and relevance of the questions and translation. After completion of the translated draft, the questionnaires were back-translated into English by an independent expert English editor. The translated version was then pilot tested among 70 participants and further refined for clarity according to their comments.

*Depression, Anxiety and Stress Scale (DASS21)*: The DASS 21 is a 21-item self-reported questionnaire designed by^25^ to measure the severity of a range of symptoms common in depression and anxiety. In completing the DASS21, the individual must indicate the presence of a symptom over the previous week. Each item scored from 0 (did not apply to me over the last week) to 3 (applied to me very much or most of the time over the past week). The essential function of the DASS21 is to assess the severity of the core symptoms of depression, anxiety, and stress.

*World Health Organization Quality of Life Instruments (WHOQOL-BREF)*: The WHOQOL-BREF is a self-administered questionnaire comprising 26 questions on the individual’s perceptions of their health and well-being over the previous two weeks. Responses to questions are on a 1–5 Likert scale. One example of an item is “How much do you enjoy life?”, rated on the following response options (1) not at all, (2) a little, (3) a moderate amount, (4) very much, and (5) an extreme amount. The WHOQOL-BREF is a shorter version of the WHOQOL-100. Both were developed by the World Health Organization (WHO) to test health and quality of life among individuals with and without diseases. High scores on WHOQOL-BREF indicate a higher degree of health and a better quality of life.

### Procedures

The research was conducted in May 2021 and targeted Palestinians in the West Bank of Palestine. The sample was recruited online using convenience sampling techniques. All participants were provided information to decide whether they wanted to participate in the research. They were provided with descriptions of the scales and the purpose of the research. Participants who agreed to participate in the research signed informed consent. The research was conducted in line with the ethical guidelines of the American Psychological Association (APA, 2010) and the Declaration of Helsinki (2013) and had been approved by the An-Najah National University IRB (Approval number: Ref: Med. May 2022/12).

### Data analysis

We used descriptive statistics, means, standard deviations, range, skewness, kurtosis, and reliability for our study variables (stress, depression, anxiety, and QoL). In addition, Person Correlation Coefficient between stress, anxiety, depression and QoL was conducted to evaluate whether there is statistical evidence for a linear relationship among our study variables. Finally, we used hierarchical regression analysis to predict stress, anxiety and depression through demographic variables (educational level, gender, region of residence, and age) in step1. Moreover, demographic variables (educational level, gender, region, and age) with the quality of life were used to predict stress, anxiety and depression in sept2. The hierarchical regression analysis has been tested using SPSS 28 software for data analysis.

### Ethics approval and consent to participate

All procedures performed in this study involving human participants were in accordance with the ethical standards of An-Najah University Research Ethics Board (IRB), the American Psychological Association (APA, 2010) and with the Helsinki Declaration (2013). Informed consent was obtained from all participants. The protocol of our study was received ethical approval from An- Najah National University Research Ethics Board (IRB) before data collection was initiated.

## Results

Descriptive statistics related to the quality of life and mental health outcomes were calculated as shown in Table 1. Participants reported high scores on quality of life, moderate scores on stress and depression, and mild scores on anxiety. Regarding internal consistency, our scales indicated a high level of reliability on Cronbach’s Alpha Formula; scores ranged from 0.84 (*stress*) to 0.91 (*QoL*).

**Table 1:** Descriptive statistics for research variables (N = 957).

Variable	Mean	S.D	Min	Max	Range	Skewness	Kurtosis	Reliability
QoL	3.3965	0.65638	1.21	4.96	3.75	– 0.514	0.215	0.91
Stress	2.1702	0.64974	1.00	4.00	3.00	0.337	– 0.077	0.84
Anxiety	1.7846	0.66828	1.00	4.00	3.00	0.957	0.607	0.87
Depression	2.1085	0.69315	1.00	4.00	3.00	0.456	– 0.236	0.85

Results of the correlational analysis are mentioned in Table 2. Specifically, QoL negatively correlated with stress (r = − 0.43*, p* < 0.01), anxiety (r = − 0.46, *p* < 0.01), and depression (r = − 0.47, *p* < 0.05). Moreover, stress positively correlated with anxiety (r = 0.81, *p* < 0.01), and depression (r = 0.89, *p* < 0.01). Finally, anxiety positively correlated with depression (r = 0.82, *p* < 0.01).

**Table 2:** Correlations among study variables (N = 957).

Measures	*1*	*2*	*3*	*4*
1.QoL	1	– 0.43**	– 0.46**	– 0.47**
2.Stress		1	0.81**	0.84**
3. Anxiety			1	0.82**
4. Depression				1

Correlation is significant at the 0.01 level (2-tailed) **.

In Table 3, we tested hierarchical regression analysis to predict stress, anxiety and depression through demographic variables (educational level, gender, region, and age) in step1. While demographic variables (educational level, gender, region, and age) with quality of life were used to predict stress, anxiety and depression in sept2. Our findings revealed that stress predicted by educational level (β = 0.15; ** *p* < *0.01*), gender, (β = 0.08; ** *p* < *0.01*), region (β = 0.07; ** *p* < *0.05*), age (β = − 0.18; ** *p* < *0.01*), and quality of life (β = − 0.40; ** p < *0.01*). Moreover, anxiety predicted by educational level (β = 0.15; ** *p* < *0.01*), gender, (β = 0.06; ** *p* < *0.05*), region (β = 0.12; ** *p* < *0.01*), age (β = 0.12; ** *p* < *0.01*), and quality of life (β = − 0.43; ** *p* < *0.01*). Finally, our findings revealed that depression predicted by educational level(β = 0.17; ** *p* < *0.01*), gender, (β = 0.09; ** *p* < *0.05*), region (β = 0.10; ** *p* < *0.01*), age (β = − 0.15; ** *p* < *0.01*), and quality of life (β = − 0.43; ** *p* < *0.01*).

**Table 3:** Hierarchical regression analysis for variables predicting stress, depression and anxiety (N = 957).

Variable	B	SEB	*β*	R2
Stress				
Step1				
Educational level	0.156	0.033	0.156**	0.10
Gender	0.126	0.044	0.089**	
Region of residence	0.103	0.040	0.079*	
Age	– 0.114	0.020	– 0.187**	
Step2				
Educational level	0.099	0.030	0.098**	0.25
Gender	0.083	0.040	0.059*	
Region of residence	0.030	0.037	0.023*	
Age	– 0.118	0.018	– 0.195**	
QoL	– 0.402	0.028	– 0.406**	
Anxiety				
Step1				
Educational level	0.164	0.034	0.158**	0.08
Gender	0.100	0.046	0.068*	
Region of residence	0.164	0.042	0.123**	
Age	– 0.077	0.021	– 0.123**	
Step2				
Educational level	0.100	0.031	0.097**	0.26
Gender	0.052	0.042	0.036*	
Region of residence	0.083	0.038	0.062*	
Age	– 0.082	0.019	– 0.131**	
QoL	– 0.442	0.029	– 0.434**	
Depression				
Step1				
Educational level	0.186	0.035	0.173**	0.10
Gender	0.144	0.047	0.095**	
Region of residence	0.145	0.043	0.104**	
Age	– 0.102	0.021	– 0.158**	
Step2				
Educational level	0.120	0.032	0.112**	0.28
Gender	0.095	0.042	0.063*	
Region of residence	0.061	0.039	0.044*	
Age	– 0.108	0.019	– 0.166**	
QoL	– 0.459	0.030	– 0.435**	

** P < *0.01;*, *p < *0.05.*

## Discussion

The current study sought to test the correlation between QoL, mental health outcomes (depression, stress, and anxiety), and the moderating effect of chosen sociodemographic factors (educational level, gender, region, and age) in the Palestinian context.

Consistent with the first study hypothesis, quality of life showed to be negatively correlated with mental health outcomes (depression, stress, and anxiety) among Palestinian adults, which also supports prior researches findings^17,19–23,26^. The individualistic perspective could explain this negative correlation, in which quality of life is subjectively assessed regarding different domains such as social, physical, and emotional well-being^4,5^. As Palestinians are experiencing challenging living conditions and different aspects of their lives, such as social bonds, psychological well-being, and economic status are compromised, it is expected that Palestinian individuals will start to suffer from psychological disorders such as depression, stress, and anxiety when assessing their lives negatively^11–14,27^.

A meta-analysis study for Marie et al.^28^ aimed to provide a syestme review of the literature concering anxity disorders and depreesive symptoms in the Palestinian context. The review of twenty-four studies from Palestine indicated that anxiety and depressive disorders are one of the most common mental health problems characterized by poor quality of life and political violence. In another study, Thabet et al.^29^ examined the correlation between war trauma, PTSD, depression and anxiety among individuals living in the Gaze strip. The results showed that war quality of life positively correlates with depression and anxiety among Palestinians.

Consistent with the second and the third study hypotheses, mental health outcomes were found to be predicted by studying sociodemographic factors and quality of life, in which educational level, gender, region, and age predicted depression, stress and anxiety, and the mentioned mental health outcomes are predicted by both the study sociodemographic factors and QoL. These findings align with previous research findings^15,16,21^. Moreover, this can be interpreted by the fact that quality of life can be affected by sociodemographic factors combined with contextual, environmental, personal, or cultural factors^30,31^, which sought to affect individuals' mental health status and mood^32^.

Our study indicated that lower levels of QoL related positively to mental health distress identified by depression, stress, and anxiety. Psychological status can be affected by the quality of life combined with sociodemographic factors. These findings are consistent with what occurs in the Palestinian context, as Palestinins people face challenging living conditions due to the ongoing military occupation that is considered a risk factor for individuals' low quality of life and psychological well-being. Allabad et al.^33^ tested the role of stressful life events in predicting poor health-related quality of life among Palestinians; the results revealed that individuals with depression or anxiety potentially face poor health-related quality of life. Massad et al.^34^ examined the correlation between health-related quality of life, trauma, stress, and depression in another study. The results indicated that Palestinians often severely impair health-related quality of life, including psychosocial health and emotional functioning. Exposure to violent and non-violent adverse events was associated with poor health-related quality of life.

### Limitations

Several limitations to our study must be noted. The results are limited to specific sociodemographic factors. The online recruitment of the sample could have limited the access to the research of the most unwell groups. In addition, this study was done using a quantitative methodology and solely relied on questionnaires and self-reports completed by the participants. It must be remembered that self-reported data may reflect no more than a tendency. It is therefore recommended to use mixed methods tools in future studies.

## Conclusion

The current study stressed the critical role of quality of life and sociodemographic factors on mental health distress among Palestinians. These findings revealed that quality of life contributes in a way that was statistically significant towards explaining variance in depression, anxiety and stress. Our results also indicated the moderator role of specific sociodemographic variables, educational level, gender, region, and age in explaining variance in depression, anxiety and depression. Improving the quality of life among Palestinians may, in one way or another, reduce anxiety, depression and stress, thus enhancing mental health and well-being outcomes among Palestinians. The results of our study highlight the positive role of mental health services in improving the quality of life and dealing with mental health distress in the Palestinian society, characterized by a high level of traumatic events and ongoing political conflict. Finally, our findings are promising to conduct other studies in order to understand better how current study variables correlate to each other, so appropriate clinical interventions to mitigate the negative effects of depression, anxiety, and stress through enhancing quality of life and positive coping Strategies can be developed and implemented by mental health providers.

## Data Availability

The datasets generated during and/or analyzed during the current study are available from the corresponding author on reasonable request.
